# Preoperative predictors of spacer retention and mortality in two-stage revision for infected total hip arthroplasties: a single-center experience of 90 patients

**DOI:** 10.1186/s42836-026-00394-7

**Published:** 2026-05-13

**Authors:** Andre Lunz, Jascha Fell, Kevin-Arno Koch, Axel Horsch, Andreas Geisbüsch, Burkhard Lehner

**Affiliations:** Department of Orthopaedics, Heidelberg University Hospital, Schlierbacher Landstrasse 200a, 69118 Heidelberg, Germany

**Keywords:** Periprosthetic joint infection, PJI, Two-stage revision arthroplasty, Mortality, Articulating hip spacer, Reimplantation, Spacer retention

## Abstract

**Background:**

Although two-stage revision arthroplasty achieves favorable infection control rates, a substantial proportion of patients experience spacer retention or short-term mortality. Therefore, the goal of this study was to identify preoperative risk factors associated with spacer retention and mortality during a two-stage approach.

**Methods:**

This single-center retrospective cohort study included a consecutive series of 90 chronic periprosthetic joint infections (PJI) of the hip treated with a two-stage revision between August 2017 and December 2021. Associations between potential risk factors and outcomes were analyzed using correlation analyses, and the effect size was quantified by Spearman's rho (r). Mortality rates were estimated using the Kaplan–Meier method and compared with the log-rank test.

**Results:**

After a mean follow-up of 48 months (range 24 to 73), 19 patients (21%) did not undergo reimplantation, 23 patients (25.5%) had died, and two patients (2%) were lost to follow-up. Successful reimplantation was significantly associated with implantation of an articulating hip spacer (*r* = 0.57) and favorable periarticular soft tissue conditions (*r* = 0.25). Mortality was significantly correlated with a higher comorbidity burden (ASA ≥ 3; *r* = 0.36) and advanced age (≥ 80 years; *r* = 0.22).

**Conclusion:**

This study identified three simple, yet powerful prognostic markers of spacer retention and mortality. Implantation of an articulating hip spacer emerged as the strongest positive predictor of successful reimplantation, although it likely functions, at least in part, as a surrogate marker for favorable local bone and soft-tissue conditions. In contrast, advanced age and a high comorbidity burden were the strongest predictors of short-term mortality. These readily assessable prognostic markers may facilitate the identification of patients at the highest risk of not completing a two-stage approach or dying shortly after.

## Introduction

Chronic periprosthetic joint infection (PJI) remains one of the most challenging complications in total joint arthroplasty. Despite substantial advances in surgical techniques and diagnostic algorithms, the two-stage approach remains the gold standard treatment for most chronic PJIs [1–4]. From a surgical perspective, durable infection control is the primary objective, and short-term eradication rates of approximately 90% are commonly reported [3, 5–10]. However, growing evidence suggests that the clinical course of patients undergoing a two-stage exchange is considerably more complex. Recent studies demonstrate that a substantial proportion of patients never proceed to reimplantation. Instead, treatment may culminate in permanent spacer retention, Girdlestone resection arthroplasty, or even amputation [7, 9–12]. Furthermore, chronic PJI is increasingly recognized as a condition with systemic health implications that extend far beyond the affected joint. Natsuhara et al. reported a five-year mortality exceeding 20% after infected total hip arthroplasty (THA) treated with a two-stage approach, representing a risk more than three times higher than the age-adjusted national expectation [13]. Complementary registry analyses have confirmed this elevated mortality risk. These studies show that PJI after primary THA is associated with a more than fivefold increase in ten-year mortality, even after adjustment for comorbidities and frailty [14]. Furthermore, a recent meta-analysis reported a one-year mortality of approximately 10% after PJI, with mortality trajectories comparable to several common malignancies, confirming the systemic burden of the disease [15].

The discrepancy between favorable infection control rates and the substantial proportion of patients with permanent spacer retention or increased mortality highlights the need for improved preoperative patient risk stratification in patients undergoing a two-stage approach. A clearer understanding of the expected clinical course may help identify patients at increased risk for adverse outcomes and support early consideration of alternative treatment strategies.

Therefore, all patients undergoing the first stage of a planned two-stage protocol for chronic hip PJI were prospectively followed. The primary objective of this study was to identify significant risk factors associated with (1) permanent spacer retention and (2) mortality during the course of two-stage revision arthroplasty.

## Material and methods

### Patient cohort

Approval was obtained from the local Medical Ethics Committee (S-443/2023). This is a retrospective cohort study investigating the course and outcome after two-stage revision arthroplasty for chronically infected THAs. Diagnosis of a chronic PJI was made in accordance with either the Musculoskeletal Infection Society [16] or the International Consensus Meeting [17, 18]. All surgeries were performed by specialized surgeons at a single tertiary referral center between August 2017 and December 2021. During the study period, our center routinely recommended a two-stage revision strategy for all patients diagnosed with chronic PJI. Only a small number of patients either declined this procedure or were deemed unsuitable candidates for a two-stage approach (e.g., patients with an ASA Score ≥ IV or peripheral arterial disease with ischemic tissue loss and necrosis of the same leg). Apart from these few cases, the study cohort includes all patients with a chronic hip PJI who were treated at our institution with a two-stage approach during the study period. Patients were eligible for inclusion, irrespective of age, comorbidities, the causative microorganism, or a prior history of septic or aseptic revision of their THA, and regardless of whether the infected THA had been implanted using cemented or cementless fixation. Exclusion criteria included acute PJIs as well as chronic PJIs treated with debridement, antibiotics, and implant retention (DAIR) or a one-stage revision. In total, 90 patients met the inclusion criteria and were included in the study. Relevant parameters regarding therapy and outcome were collected based on the prospectively organized institutional arthroplasty registry and a prospective follow-up was conducted. To identify potential risk factors associated with spacer retention and mortality, correlations between these two outcomes and perioperative parameters were analyzed.

### Surgical technique and treatment course

Treatment regimen and postoperative care were performed in accordance with recently published recommendations [19–21]. Although many implants become loosened due to chronic infection, the removal of well-fixed stems can be technically demanding during first-stage surgery. In our center, femoral stems are primarily removed using an endofemoral approach, with only two patients requiring a transfemoral osteotomy in this cohort. Removal of cemented stems is generally straightforward; however, extraction of the cement mantle can be more challenging. For this purpose, we routinely use the TORS system (Torsional Orthopaedic Revision System, Endocon GmbH, Wiesenbach, Germany), an ultrasonic device for efficient bone cement removal. Most cementless stems can likewise be removed using an endofemoral approach, following meticulous exposure of the prosthetic shoulder and stepwise disruption of the bone-implant interface. In difficult cases, we routinely employ the OrthoClast system (Endocon GmbH, Wiesenbach, Germany), which uses pneumatic shockwave technology to facilitate minimally invasive implant mobilization. Femoral fissures were rare and were successfully treated with one or two cerclage wires and postoperative protected weight-bearing. After complete implant removal, at least five tissue samples were obtained for microbiological and histological analysis, followed by thorough surgical debridement and extensive irrigation. Based on the surgeons’ discretion and intraoperative findings, either a custom-made articulating hip spacer was inserted or a resection arthroplasty (Girdlestone) with antibiotic-loaded bone cement filling of the proximal femur and acetabulum was performed [22, 23]. A commercially available antibiotic-loaded bone cement (Palacos R + G, Heraeus Medical GmbH, Wehrheim, Germany) is routinely used for spacer fabrication. In addition, a second pathogen-specific antibiotic is incorporated into the cement, most commonly vancomycin (3–4 g per 40 g cement). The surgical technique for intraoperative construction of the custom-made articulating hip spacer has been described and published previously [24, 25]. For the interim period, patients with articulating hip spacers were advised to perform partial weight-bearing to prevent spacer-related mechanical complications, while all other patients were advised to perform no weight-bearing. Empirical, broad-spectrum intravenous antibiotic therapy was administered, and de-escalation to a targeted therapy was performed as soon as microbiological results were available. In case of culture-negative PJIs, a broad empiric antibiotic treatment was maintained throughout the interim period and after second-stage surgery. Reimplantation was performed once eradication of infection was indicated by both clinical and laboratory criteria, irrespective of the causative pathogen. Clinical criteria included complete wound healing, absence of sinus tract formation, and no signs of local inflammation. Laboratory criteria comprised normalization of white blood cell count and C-reactive protein levels. Systemic antibiotic therapy was typically continued for six weeks after reimplantation, with regular follow-ups conducted by primary care physicians and at our outpatient clinics.

### Data analyses

Descriptive statistics are presented as numbers of occurrences, percentages, or arithmetic means, and ranges or standard deviations (SD). Shapiro–Wilk tests were used to assess normality. Kaplan–Meier survivorship analyses were used to determine survival rates for different endpoints. Differences in survival rates between groups were tested for statistical significance using two-sided log-rank (Mantel–Cox) tests. Hazard ratios for the risk of revision, with 95% confidence intervals (95% CIs), were calculated using the Mantel–Haenszel method. To assess the association between mortality or failure to progress to reimplantation and perioperative parameters, Spearman’s rank correlation and chi-square test were conducted. In case of a statistically significant association, the effect size was calculated by determining Spearman’s rho (r). The effect size is defined as small (0.1), medium (0.3), or large (0.5). The level of significance was set at *p* < 0.05 for all statistical tests, and the statistical analyses were performed using Statistical Package for Social Sciences (SPSS) (version 27.0; IBM, Armonk, New York, USA) and GraphPad Prism 8 (GraphPad Prism; GraphPad Software, San Diego, California, USA).

## Results

A total of 90 consecutive patients with 90 chronically infected THAs were included in this study. A comprehensive summary of the patient demographics and microbiological results is given in Table 1. All patients were diagnosed with a chronic PJI of their THA, and treatment was started with the intention to perform a two-stage revision arthroplasty. All patients underwent first-stage surgery with either implantation of a custom-made articulating hip spacer (*n* = 57, [63%]), or performance of a Girdlestone resection arthroplasty (*n* = 33, [37%]) with cement filling of the proximal femur and acetabulum.

**Table 1:** Summary of patient demographics and microbiological results

**Variable**	**Mean ± SD or *n* (%)**
**Demographics**	
Study cohort	90 (100%)
Age at first-stage surgery (years)	70 ± 9
Female sex	36 (40%)
CCI (age-adjusted)	3.3 ± 1.5
ASA	2.6 ± 0.7
**Comorbidities,** **most common,** *n* (%)	
COPD	14 (16%)
Heart failure/Myocardial infarction	13 (14%)
Diabetes mellitus	11 (12%)
Malignancy	9 (10%)
Peripheral arterial disease	7 (8%)
**Characteristics of infected THAs**	
Previous septic revision	37 (41%)
Cementless fixation of THA	58 (64%)
Septic loosening of THA	43 (48%)
**Microbiological results**	
**Number of organisms,** *n* (%)	
Culture-negative infection	17 (19%)
Monomicrobial	51 (57%)
Polymicrobial	22 (24%)
**Most common organisms,** *n* (%)	
CoNS	43 (48%)
Staphylococcus aureus	17 (19%)
Gram-negative bacteria	11 (12%)
Streptococcus species	10 (11%)
Cutibacterium species	8 (9%)

The values are given as either mean ± standard deviation (SD) or number (n) with percentage in brackets (%)

*CCI* Charlsen Comorbidity Index, *ASA* American Society of Anesthesiologists Physical Status Classification, *COPD* Chronic obstructive pulmonary disease, *THA *Total Hip Arthroplasty*,* *CoNS* Coagulase Negative Staphylococcus

**Fig. 1:**
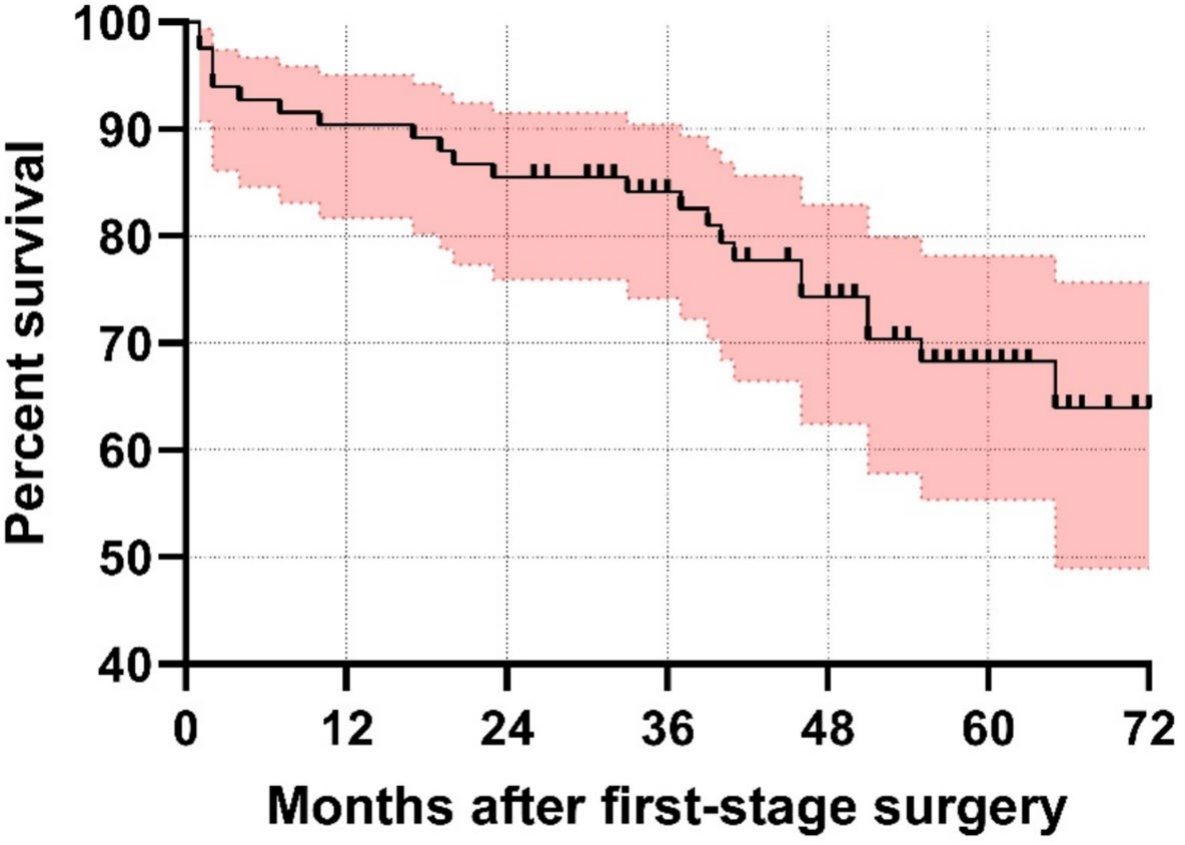
Kaplan–Meier survivorship curve for death as endpoint. The one- and five-year mortality rates were estimated at 10% (95% CI, 5–18) and 32% (95% CI, 21–45), respectively

The mean follow-up time was 48 months (range, 24 to 73). There were two patients (2%) lost to follow-up after first-stage surgery. After a mean interim period of 97 days (range, 17 to 365), a total of 69 patients (77%) progressed to second-stage surgery with reimplantation of a new THA. The remaining 19 patients did not proceed to reimplantation: 9 patients (10%) were assessed as medically unfit to undergo reimplantation, 6 (7%) had deceased prior to the planned reimplantation, and 4 (4%) had signs of a persistent infection with poor soft tissues or bone stock. At final follow-up, a total of 23 patients had died, resulting in one-year and five-year mortality rates of 10% and 32%, respectively. The Kaplan–Meier survivorship curve with the endpoint “death” is shown in Fig. 1.

### Correlation between perioperative parameters and spacer retention

A statistically significant correlation with a large effect size was identified between reimplantation and implantation of articulating hip spacers at first-stage surgery (Spearman’s rho (*r*) = 0.57). Furthermore, statistically significant correlations with a medium effect size were identified between reimplantation and good preoperative soft tissues around the affected joint (*r* = 0.25) and patients’ preoperative comorbidity level assessed either with the American Society of Anesthesiologists Physical Status Classification System (ASA) (*r* = 0.28) or the Charlson Comorbidity Index (CCI) (*r* = 0.16). Unplanned surgical revisions during the interim period showed no significant correlation with failure to finally progress to reimplantation (*r* = 0.08). The results are summarized in Table 2.

**Table 2:** Correlations between potential risk factors and spacer retention or mortality

**Potential risk factor**	**Correlation* r***	
	**Spacer retention (*n* = 19)**	**Mortality (*n* = 23)**
Non-articulating hip spacer	***r* = 0.57**	***r* = 0.51**
Condition of soft tissues around the affected joint	***r* = 0.25**	*r* = 0.19
Comorbidity level – ASA	***r* = 0.28**	***r* = 0.36**
Comorbidity level – CCI	*r* = 0.16	***r* = 0.39**
Patient age at first-stage surgery	*r* = 0.06	***r* = 0.22**
Patient BMI at first-stage surgery	*r* = 0.03	*r* = 0.04
Implant type (standard, revision, megaprosthesis)	*r* = 0.04	*r* = 0.13
Spacer revision during the interim period	*r* = 0.08	*r* = 0.08

To assess correlations, Spearman’s rank and chi-square test were conducted. In case of a statistically significant association, the effect size (small ≈ 0.1, medium ≈ 0.3, or large ≈ 0.5) was calculated by determining Spearman’s rho (r). Bold formatting was applied if the correlation analysis reached a medium or large effect size (*r* > 0.2)

*CCI* Charlson Comorbidity Index, *ASA* American Society of Anesthesiologists Physical Status Classification, *BMI* Body Mass Index

### Correlation between perioperative parameters and mortality

Statistically significant correlations with a medium-to-large effect size were identified between mortality and preoperative ASA (*r* = 0.36) and CCI (*r* = 0.39). Furthermore, there was a statistically significant association with a medium effect size between mortality and patient age at first-stage surgery (*r* = 0.22). The results are summarized in Table 2.

**Fig. 2:**
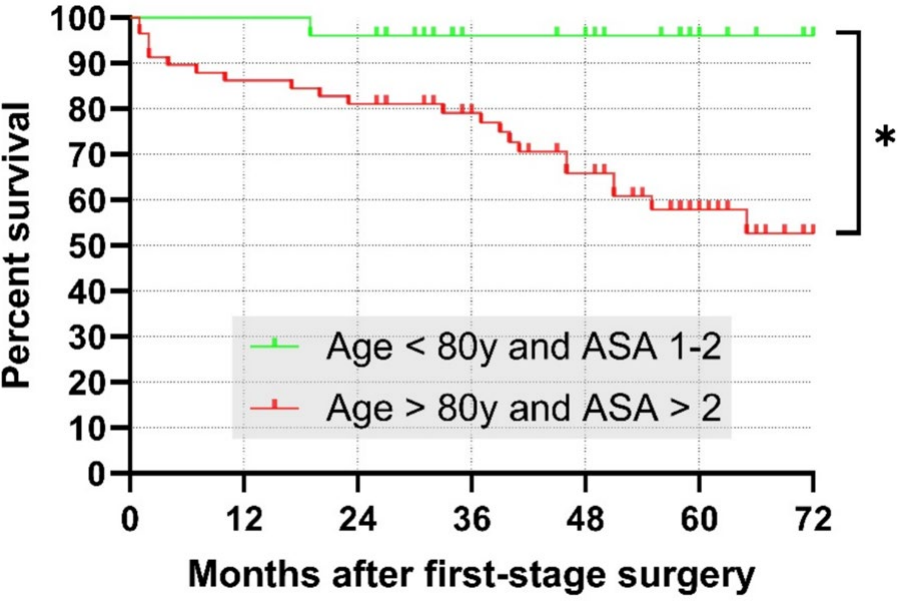
Kaplan–Meier survivorship curves for death as endpoint. The estimated one- and five-year mortality rates were 0% and 4% in the low-risk group, and 14% and 42% in the high-risk group, respectively. Survival differed significantly between groups (log-rank [Mantel–Cox] test, *p* = 0.0043). “low-risk” group (green): age < 80 years and ASA 1–2; “high-risk” group (red): age ≥ 80 years and ASA 3

Based on the identified correlations, two patient cohorts were defined. The first group included patients with an ASA score of 1–2 and an age under 80 years. The second group consisted of patients with an ASA score of 3 and age greater than 80 years. Kaplan–Meier survivorship analyses were performed for the endpoint mortality in both groups: the estimated one- and five-year mortality rates were 0% and 4% in the low-risk group, and 14% and 42% in the high-risk group, respectively. (Fig. 2). Comparison of the two survival curves showed a statistically significant difference (Log-rank test, *p* = 0.0043).

## Discussion

**Fig. 3:**
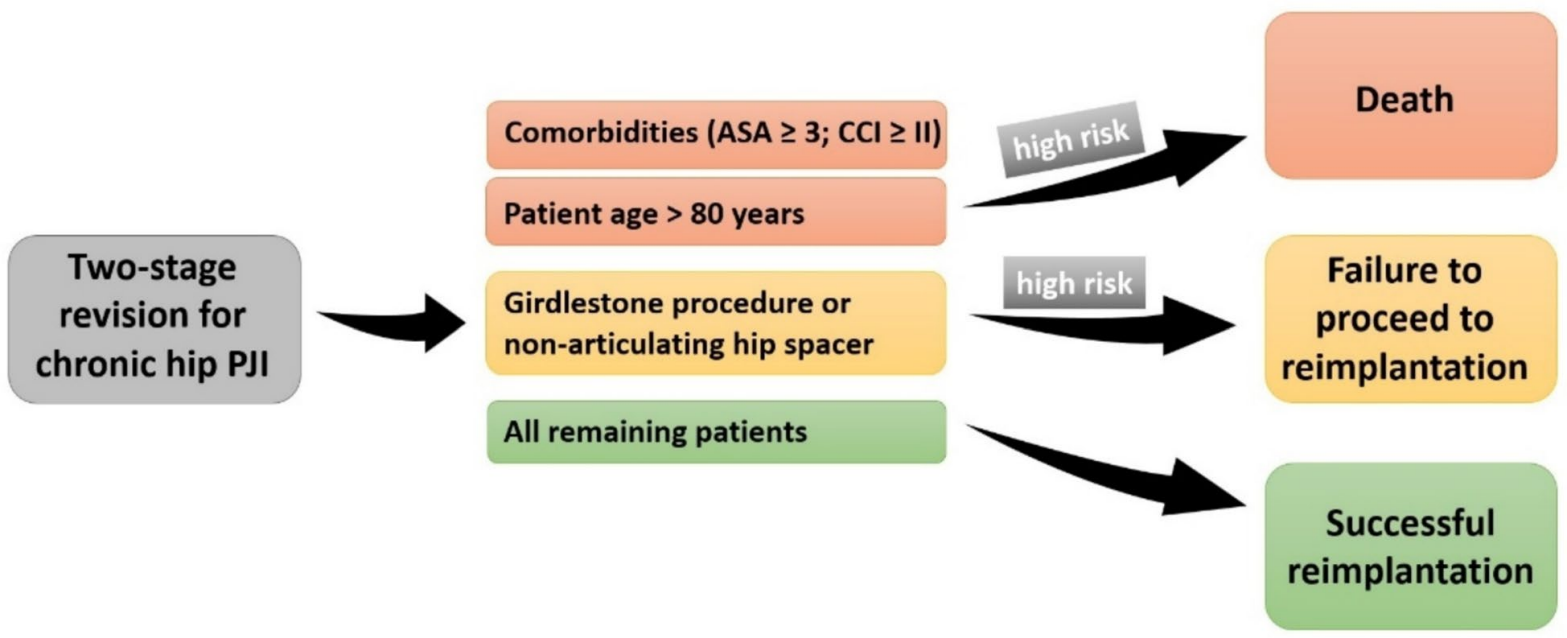
Two-stage revision arthroplasty for chronic periprosthetic joint infection of the hip results in three possible short-term outcomes: death, spacer retention, or successful reimplantation. Preoperative ASA score, patient age, and type of hip spacer were identified as strong prognostic markers. A high comorbidity burden (ASA ≥ 3 or CCI ≥ II) and advanced age (≥ 80 years) were associated with a significantly increased risk of short-term mortality. Patients treated with Girdlestone resection arthroplasty or implantation of a non-articulating hip spacer during first-stage surgery demonstrated a high probability of permanent spacer retention

Two-stage exchange arthroplasty is widely regarded as the gold standard for the treatment of chronic PJIs, achieving favorable short-term infection control rates [3, 4]. At the same time, a considerable proportion of patients either do not proceed to reimplantation after first-stage surgery or die during the course of two-stage revision [10–12, 26]. Therefore, reliable preoperative prediction of the most likely outcome (death, spacer retention, or successful reimplantation) is of paramount importance. This study identified three simple, yet powerful prognostic markers of spacer retention and mortality (Fig. 3).

**Fig. 4:**
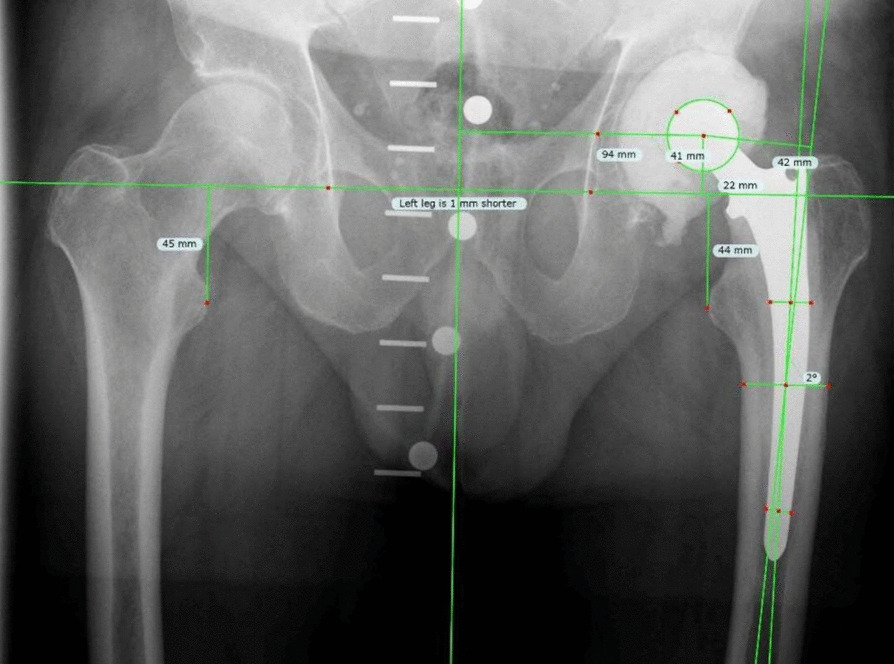
Anteroposterior pelvic radiograph demonstrating a precise restoration of patient-specific hip biomechanics after implantation of the custom-made articulating hip spacer. The custom-made spacer allows accurate reconstruction of femoral offset and center of rotation, resulting in near-symmetric limb length and physiological alignment. This is essential to maintain soft-tissue tension, optimize function, reduce instability, and facilitate subsequent second-stage reimplantation

The spacer retention rate of approximately 20% observed in this study is consistent with rates reported in recent literature and has remained largely unchanged over the past decade [11, 27]. Most affected patients ultimately experience permanent immobilization and frequently require additional surgical procedures, including spacer exchange or even amputation. Therefore, reliable preoperative identification of patients at high risk for definitive spacer retention during a planned two-stage approach is essential. Such risk stratification would allow reconsideration of the treatment strategy and facilitate informed decision-making regarding alternative treatment options. In the present study, implantation of an articulating hip spacer at first-stage surgery was the strongest predictor for successful completion of the two-stage protocol, although it likely functions, at least in part, as a surrogate marker for more favorable local bone and soft-tissue conditions (Fig. 4). The custom-made articulating hip spacer demonstrated a spacer retention rate of only 3.5%, compared with 51.5% following Girdlestone resection or implantation of a non-articulating hip spacer. Conversely, patients who are not suitable candidates for an articulating hip spacer should be counseled regarding their substantially increased risk of definitive spacer retention, and alternative treatment strategies should be considered. Further studies are warranted to validate these findings and to identify additional preoperative predictors.

**Fig. 5:**
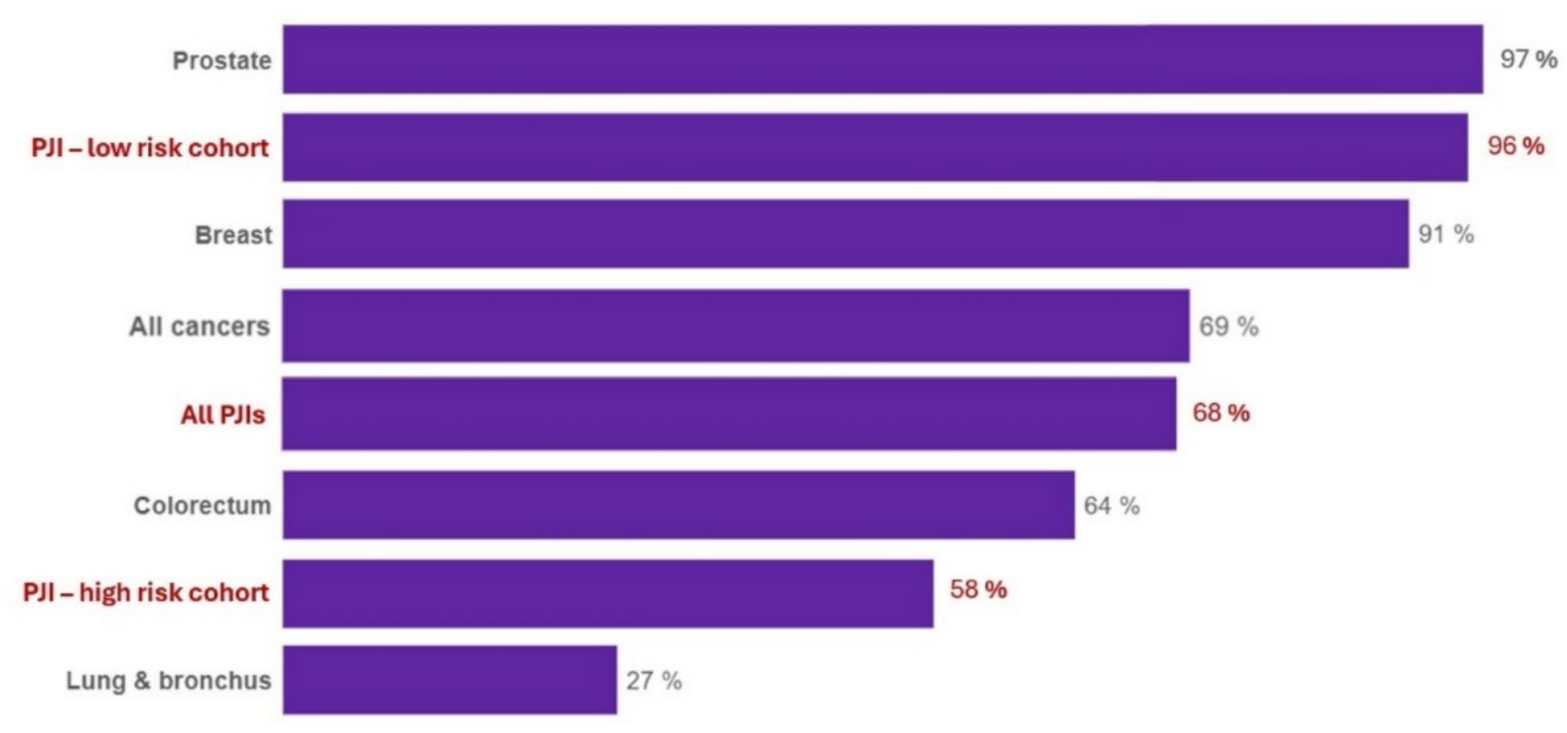
Bar chart illustrating five-year patient survival rates of selected common malignancies compared with chronic periprosthetic joint infection (PJI). PJI - low-risk cohort: age < 80 years and ASA 1–2; PJI - high-risk cohort: age ≥ 80 years and ASA 3

The one- and five-year mortality rates observed in this study (10 and 32%, respectively) are consistent with the results of a recent meta-analysis by Ramos et al. [15]. Similarly, Zmistowski et al. reported significantly higher mortality after septic revision arthroplasty compared with aseptic revision, with one- and five-year mortality rates of 10.6 and 25.9%, respectively [28]. Comparable findings have been described in several other studies [12, 13, 26]. These observations highlight the systemic and debilitating nature of PJI and suggest that mortality associated with chronic PJI is comparable to that reported for common malignancies (Fig. 5) [29]. In analogy to oncology, the identification of reliable prognostic markers is therefore essential to estimate the individual risk of mortality. This study identified patient age and comorbidity burden as strong prognostic markers of mortality. Based on the derived cut-off values, two risk groups were defined: a low-risk group (< 80 years and ASA ≤ 2) and a high-risk group (≥ 80 years and ASA ≥ 3). Mortality differed significantly between these groups (*p* < 0.0043). The estimated one- and five-year mortality rates were 0 and 4% in the low-risk group, compared to 14 and 42% in the high-risk group (Fig. 2). These findings suggest that patients preoperatively classified as high risk based on age and comorbidity burden should be counseled carefully regarding their substantial mortality risk when undergoing a two-stage approach. Further studies are required to validate these findings and to identify additional pre-operative predictors of mortality.

This study has potential limitations. It is a retrospective study with a mean follow-up of four years and inclusion of 90 consecutive patients. Due to the low incidence of chronic PJIs in general, single-center studies rarely include substantially larger patient numbers [12, 30, 31]. Therefore, transferability of the results to other patients or institutions is limited, and all identified associations have no proven causality. However, a longer follow-up period was not required to address the study objectives, as it would not alter reimplantation rates or short-term mortality rates. This study focused exclusively on chronically infected THAs during a contemporary period at a single specialized center. During the study period, all patients were treated according to the same two-stage protocol, and no patients were excluded. Consequently, this study presents real-world data of a non-selected patient cohort treated with a two-stage approach according to recent recommendations [19].

## Conclusions

Although two-stage revision arthroplasty achieves high infection control rates, a substantial proportion of patients experience spacer retention or short-term mortality. This study identified three simple, yet powerful prognostic markers of spacer retention and mortality. Implantation of an articulating hip spacer emerged as the strongest positive predictor of successful reimplantation, although it likely functions, at least in part, as a surrogate marker for more favorable local bone and soft-tissue conditions. In contrast, advanced age (≥ 80 years) and a high comorbidity burden (ASA ≥ 3) were the strongest prognostic markers of short-term mortality. These findings may support clinical decision-making and help guide counseling of affected patients toward alternative treatment strategies, thereby potentially preventing catastrophic outcomes.

## Data Availability

The datasets generated and/or analyzed during the current study are not publicly available to preserve individuals’ privacy under the European General Data Protection Regulation but are available from the corresponding author on reasonable request.

## Abbreviations

ASA: American Society of Anesthesiologists Physical Status Classification System
CCI: Charlson Comorbidity Index
CI: Confidence Interval
COPD: Chronic Obstructive Pulmonary Disease
DAIR: Debridement, Antibiotics, and Implant Retention
K-M: Kaplan–Meier Survivorship
PJI: Periprosthetic Joint Infection
PROM: Patient-reported Outcome Measure
r: Spearman’s rho
SD: Standard Deviation
UCLA: University of California, Los Angeles Activity Score
THA: Total Hip Arthroplasty
VAS: Visual Analog Scale
